# Impact of Platelet Endothelial Aggregation Receptor-1 Genotypes on Long-Term Cerebrovascular Outcomes in Patients With Minor Stroke or Transient Ischemic Attack

**DOI:** 10.3389/fneur.2021.649056

**Published:** 2021-05-28

**Authors:** Xiao-Guang Zhang, Jing-Yu Gu, Qiang-Qiang Fu, Shi-Wu Chen, Jie Xue, Shan-Shan Jiang, Yu-Ming Kong, You-Mei Li, Yun-Hua Yue

**Affiliations:** Department of Neurology, Yangpu Hospital, Tongji University School of Medicine, Shanghai, China

**Keywords:** acute minor ischemic stroke, transient ischemic attack, PEAR1, genetic polymorphism, cerebrovascular outcomes

## Abstract

**Background:** Platelet endothelial aggregation receptor-1 (PEAR1) rs12041331 has been reported to affect agonist-stimulated platelet aggregation, but it remains unclear whether this variant plays a role in recurrent stroke. Here we assess the clinical relevance of PEAR1 rs12041331 in acute minor ischemic stroke (AMIS) and transient ischemic attack (TIA) Chinese patients treated with dual antiplatelet therapy (DAPT).

**Methods:** We recruited 273 consecutive minor stroke and TIA patients, and Cox proportional hazard regression was used to model the relationship between PEAR1 rs12041331 and thrombotic and bleeding events.

**Results:** Genotyping for PEAR1 rs12041331 showed 49 (18.0%) AA homozygotes, 129 (47.3%) GA heterozygotes, and 95 (34.7%) GG homozygotes. No association was observed between PEAR1 rs12041331 genotype and stroke or composite clinical vascular event rates (ischemic stroke, hemorrhagic stroke, TIA, myocardial infarction, or vascular death) or bleeding events regardless if individuals carried one or two copies of the A allele. Our results suggested that rs12041331 genetic polymorphism was not an important contributor to clinical events in AMIS and TIA patients in the setting of secondary prevention.

**Conclusions:** Our data do provide robust evidence that genetic variation in PEAR1 rs12041331 do not contribute to atherothrombotic or bleeding risk in minor stroke and TIA patients treated with DAPT.

## Introduction

Patients with acute minor ischemic stroke (AMIS) and transient ischemic attack (TIA) have a high risk of recurrent stroke and cardiovascular events (1). Current guidelines recommend dual antiplatelet therapy (DAPT) with aspirin and clopidogrel as a standard of care for patients with AMIS and TIA who can be treated within 24 h after the onset of symptoms (2, 3). However, CHANCE trial reported that up to 8.2% of patients receiving DAPT still experienced a recurrent stroke (2). Antiplatelet drug resistance was found to contribute to recurrent stroke (4, 5), which may be associated with many potential genetic and environmental factors (6). However, the correlation between genetic polymorphisms and recurrent stroke is not yet fully elaborated.

Platelet endothelial aggregation receptor-1 (PEAR1) is a platelet transmembrane tyrosine kinase receptor involved in platelet–platelet contact and platelet aggregation, which is highly expressed in platelets and endothelium (7). Studies have shown that genetic variants in the PEAR1 gene were not only associated with platelet aggregation (8, 9) but also the response to antiplatelet agents including aspirin (10) and clopidogrel (11). Rs12041331 is an intronic variant that is supposed to modify PEAR1 expression in the protein level *via* a different allele-specific DNA methylation and has been found to be correlated with platelet aggregation (12, 13). Although there has been evidence that PEAR1 rs12041331 has an effect on agonist-stimulated platelet aggregation in aspirin-treated patients (14), it is still controversial whether this genetic polymorphism in PEAR1 is associated with clinical outcomes. Xu et al. reported that PEAR1 rs12041331 plays an important role in early cardiovascular outcomes in patients undergoing percutaneous coronary intervention in a Chinese population (15). However, in a white population, Yang et al. could not replicate previous reports from experimental studies or obtained in patients suggesting that PEAR1 might be a susceptibility gene for cardiovascular complications (16). Currently, research on the relationship between PEAR1 rs12041331 and the clinical events were mainly focused on coronary heart diseases. In a retrospective, case–control study, the A allele showed a higher frequency than the G allele in the recurrent ischemic stroke group (17). However, there has been no study on the relationship between PEAR1 polymorphism and the long-term cerebrovascular events in patients with minor stroke and TIA.

To assess the clinical relevance of PEAR1 rs12041331 in Chinese AMIS and TIA patients treated with DAPT, we investigated the prevalence of PEAR1 rs12041331 genotypes and estimated its association with long-term cerebrovascular events, bleeding events, and clinical function.

## Method

### Study Population

We conducted a single-center cohort study based on data collected from April 2016 to December 2018 from 273 AMIS and TIA patients in the Department of Neurology of the Yangpu Hospital, Tongji University School of Medicine. The collection and genetic analysis of samples were approved by the ethics committee of Yangpu Hospital, Tongji University School of Medicine (Ethical Approval Number LL-2016-SCI-001). Informed consent has been obtained. The study enrolled patients who were at least 40 years of age and had an AMIS, with a National Institutes of Health Stroke Scale score of ≤ 3 on admission (range, 0–42, with higher scores indicating a more severe stroke), or those with a moderate to high risk of TIA according to an ABCD^2^ stroke risk score of ≥4 on admission (range, 0–7, with higher scores indicating a higher risk of stroke) or ≥50% stenosis of cervical or intracranial vessels that could account for the presentation who could be treated with DAPT (100 mg aspirin and 75 mg clopidogrel, once daily for 21 days) within 24 h of symptom onset. Patients were excluded from the study if they had hemorrhage on baseline brain computed tomography (CT) or another pathology that could account for the neurological symptoms or had a contraindication to aspirin or clopidogrel.

### Outcomes

We analyzed the relationship between the common variant PEAR1 rs12041331 and the clinically adjudicated long-term cerebrovascular events, bleeding events, and clinical function after DAPT application. The primary efficacy endpoint for this trial was a new stroke event (ischemic or hemorrhagic) that happens within 2 years. Ischemic stroke is defined as a sudden focal neurological dysfunction caused by vascular causes with duration ≥24 h or a neurological dysfunction due to imaging and clinical symptoms caused by bloody infarction rather than cerebral hemorrhage found by imaging examination. Hemorrhagic stroke is defined as the acute extravasation of blood into the brain parenchyma or subarachnoid space with associated neurological symptoms. A diagnosis of stroke should be confirmed by neuroimaging (CT or MRI).

The secondary efficacy endpoint was analyzed as the individual or composite outcomes of the new clinical vascular event (ischemic stroke, hemorrhagic stroke, TIA, myocardial infarction, or vascular death). Vascular death is defined as death resulting from stroke (ischemic or hemorrhagic), systemic hemorrhage, myocardial infarction, congestive heart failure, pulmonary embolism, sudden death, or arrhythmia. If multiple vascular events occurred in the same patient during the follow-up period, the composite event was counted as one person time.

A safety endpoint included intracranial hemorrhage and bleeding of any other cause. Bleeding events were classified as major bleeding (a decrease in hemoglobin level of 2 g/dl or greater within a 24-h period or leading to a transfusion of two or more units of packed red cells or requiring an additional intervention) or minor bleeding according to the International Society on Thrombosis and Hemostasis criteria (18).

The study included one visit, which was 2 years (1 month either way) after the start of DAPT. Face-to-face or telephone interviews were involved in all visits, with data collected on electronic case report forms.

### Genetic Analysis

Genomic DNA was extracted from whole blood samples (empty stomach on the early morning of the day after admission) with a Lab-Aid nucleic acid (DNA) magnetic bead separation kit (Zeesan, Xiamen) according to the manufacturer's instructions. The PEAR1 rs12041331 in human whole blood genomic DNA was detected by the combination of multiplex allele-specific PCR and universal array developed by CapitalBio Technology Corporation, Ltd. Using human whole blood genomic DNA as the template, amplicons from PEAR1 gene were multiplex PCR-amplified with allele-specific PCR primers. After amplification, the reaction mixture was hybridized with specific tag probes immobilized on a microarray chip in the CapitalBio BioMixerTM II Microarray Hybridization Station (CapitalBio Corporation, Beijing, China). Hybridization was stopped by washing the slide in a wash buffer. The chips were scanned and imaged using LuxScan 10K-B Microarray Scanner (CapitalBio Corporation, Beijing, China). The detection results of polymorphic loci were obtained.

### Statistical Analysis

Continuous variables were described as median (interquartile intervals, 25–75), categorical variables were expressed as frequencies and percentages, and differences of the baseline characteristics among AA, GA, and GG genotypes of PEAR1 rs12041331 were assessed by one-way ANOVA (for continuous variables) or χ^2^-test (for categorical variables). Cox proportional hazard models were adopted to perform a primary analysis comparing the cumulative incidences of 2-year cerebrovascular events among patients with AA, GA, and GG genotypes of PEAR1 rs12041331. The results were presented as hazard ratio (HR) with 95% CI.

The statistical analysis was carried out using SPSS, version 21.0 (IBM Corporation, Armonk, NY, USA) and Stata, version 15.0 (Stata Corporation, College Station, TX, USA) statistical software. All tests were two-sided, and *P* < 0.05 was considered statistically significant.

## Results

### Baseline Characteristics of Patients by PEAR1 Genotypes

**Figure 1:**
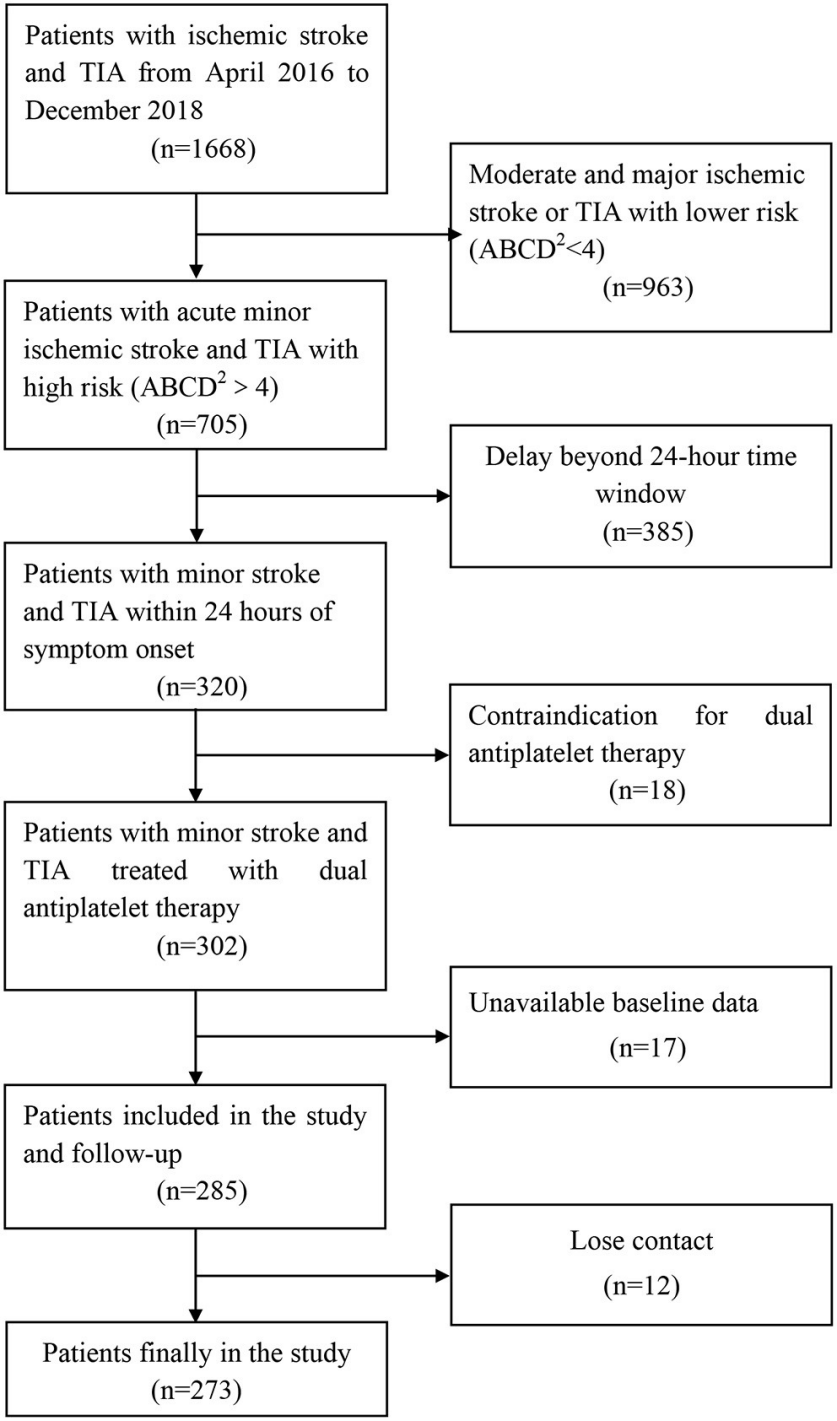
Trial profile. TIA, transient ischemic attack.

From April 2016 to December 2018, a total of 1,668 patients were involved, of whom 320 had minor stroke and TIA within 24 h of symptom onset. A total of 18 patients had contraindication for DAPT during the treatment period, 17 patients had unavailable baseline data, and 12 patients lost contact during the follow-up (Figure 1). Overall, 273 patients were enrolled and contributed samples for genotyping in this study. The baseline characteristics were similar among the groups (Table 1). Most patients (89%) presented with minor stroke, and 11% patients presented with TIA. The median age of the patients was 66 years old, and 70% were men. The mean BMI of the patients was 24 kg/m^2^. Most patients continued to use aspirin after 21-day DAPT. Genotyping for PEAR1 rs12041331 showed 49 (18.0%) AA homozygotes, 129 (47.2%) GA heterozygotes, 178 (65.2%) AA + GA heterozygotes, and 95 (34.7%) GG homozygotes. Patients with the GG homozygotes have a higher history of stroke. The distributions of age, sex, BMI, vascular risk factors, NHISS, mRS, TOAST, and laboratory data variables were not statistically different among the groups.

**Table 1:** Baseline characteristics of patients with different PEAR1 genotypes.

**Characteristics**	**AA (*n* = 49)**	**GA (*n* = 129)**	**GG (*n* = 95)**	***P*-value**	**AA + GA (*n* = 178)**	**GG (*n* = 95)**	***P*-value**
Age, years	65 (61–76.5)	66 (59–77)	67 (62–80)	0.815	66 (59.75–77)	67 (62–80)	0.217
Male sex, *n* (%)	34 (69.4)	88 (68.2)	69 (72.6)	0.772	122 (68.5)	69 (72.6)	0.482
Body mass index, kg/m^2^	23.94 (22.48–26.76)	24.49 (22.45–25.95)	24.44 (22.60–25.95)	0.853	24.42 (22.47–26.06)	24.44 (22.60–25.95)	0.560
Diagnosis, *n* (%)				0.388			0.298
Minor stroke	46 (93.9)	115 (89.1)	82 (86.3)		161 (90.4)	82 (86.3)	
TIA	3 (6.1)	14 (10.9)	13 (13.7)		17 (9.6)	13 (13.7)	
Hypertension, *n* (%)	41 (83.7)	101 (78.3)	70 (73.7)	0.384	142 (79.8)	70 (73.7)	0.250
Diabetes mellitus, *n* (%)	22 (44.9)	51 (39.5)	48 (50.5)	0.261	73 (41)	48 (50.5)	0.132
Previous stroke, *n* (%)	5 (10.2)	13 (10.1)	23 (24.2)	0.008	18 (10.1)	23 (24.2)	0.002
Previous coronary artery disease, *n* (%)	2 (4.1)	10 (7.8)	10 (10.5)	0.398	12 (6.7)	10 (10.5)	0.274
Previous or current smoker, *n* (%)	25 (51.0)	60 (46.5)	42 (44.2)	0.74	85 (47.8)	42 (44.2)	0.576
Alcohol, *n* (%)	8 (16.3)	23 (17.8)	17 (17.9)	0.968	31 (17.4)	17 (17.9)	0.921
NIHSS	1 (0–2)	1 (0–2)	1 (0–3)	0.502	1 (0–2)	1 (0–3)	0.401
mRS	1 (0.5–2)	1 (0–2)	1 (0–2)	0.73	1 (0–2)	1 (0–2)	0.833
TOAST				0.681			0.969
Large artery atherosclerosis	23 (46.9)	49 (38.0)	40 (42.1)		72 (40.4)	40 (42.1)	
Cardioaortic embolism	0	7 (5.4)	5 (5.3)		7 (3.9)	5 (5.3)	
Small artery occlusion	24 (49.0)	63 (48.8)	45 (47.3)		87 (48.9)	45 (47.3)	
Other causes	2 (4.1)	5 (3.9)	3 (3.2)		7 (3.9)	3 (3.2)	
Undetermined causes	0	5 (3.9)	2 (2.1)		5 (2.8)	2 (2.1)	
Creatinine, μmol/L	74.73 ± 26.19	81.24 ± 34.37	82.13 ± 37.67	0.478	79.45 ± 32.39	82.13 ± 37.67	0.540
Glucose, mmol/L	7.27 ± 3.51	6.88 ± 3.05	7.65 ± 4.85	0.057	6.99 ± 3.18	7.65 ± 4.85	0.177
HDL-C, mmol/L	1.01 ± 0.24	1.03 ± 0.27	1.10 ± 0.31	0.113	1.02 ± 0.27	1.10 ± 0.31	0.057
LDL-C, mmol/L	3.20 ± 0.65	2.97 ± 0.85	3.07 ± 1.04	0.059	3.03 ± 0.81	3.07 ± 1.04	0.751
Antiplatelet drugs after 21 days				0.472			0.374
Aspirin	45	123	87		168	87	
Clopidogrel	4	6	8		10	8	

*Values are presented as mean ± SD or number of patients (percentage) as appropriate. P-values represent the statistical difference of each variable by PEAR1 rs12041331 genotype*.

*TIA, transient ischemic attack; NIHSS, National Institutes of Health Stroke Scale; mRS, modified Rankin scale; TOAST, trial of org 10,172 in acute stroke treatment; HDL-C, high-density lipoprotein cholesterol; LDL-C, low-density lipoprotein cholesterol*.

### Efficacy Outcomes and Genotypes

**Figure 2:**
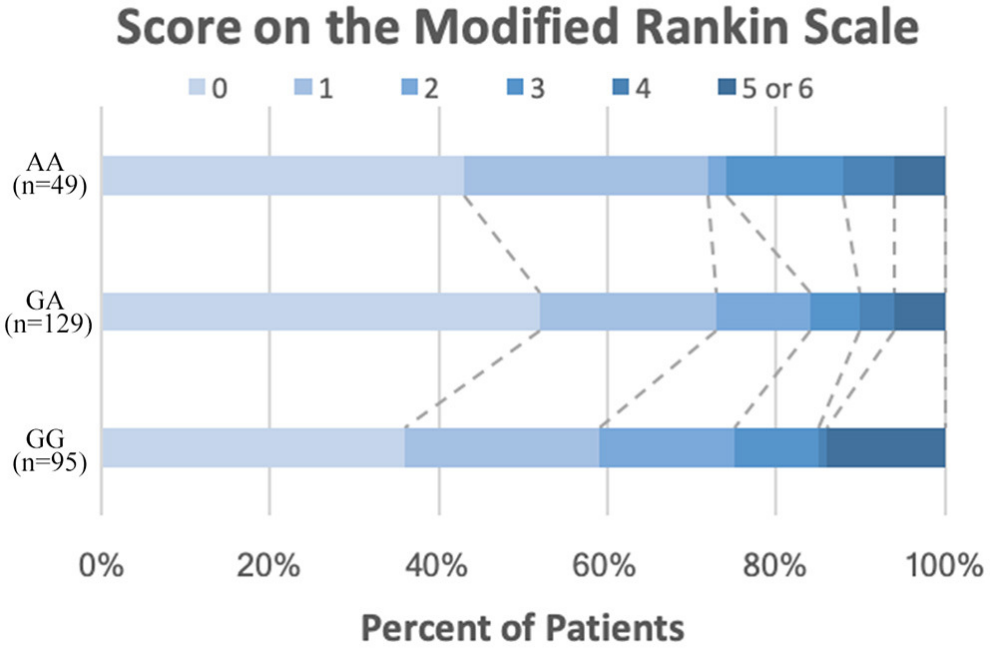
Distribution of scores on the modified Rankin scale at 2 years. The distribution of scores for disability on the modified Rankin scale among patients by different PEAR1 rs12041331 genotype ranges from 0 to 6, with higher scores indicating more severe disability.

All patients completed the 2-year clinical follow-up. The primary outcome (stroke) was observed in 23 of 273 patients (8.42%), and the secondary efficacy outcomes (composite clinical vascular events) occurred in 24 patients (8.79%). The median score for disability on the modified Rankin scale (mRS) at 2 years was 1 in the AA homozygotes, 0 in the GA homozygotes, and 1 in the GG homozygotes of PEAR1 rs12041331 (Figure 2 and Table 2).

**Table 2:** Association of PEAR1 rs12041331 with cerebrovascular events within 2 years.

**Vascular events**	**AA (*n* = 49)**	**GA (*n* = 129)**	**GG (*n* = 95)**	**AA vs. GG**		**GA vs. GG**		**AA** **+** **GA vs. GG**	
				**HR 95% CI**	***P***	**HR 95% CI**	***P***	**HR 95% CI**	***P***
**Primary efficacy outcomes**									
Stroke	6	11	6	2.00 (0.65–6.21)	0.229	1.36 (0.50–3.68)	0.545	1.53 (0.60–3.89)	0.368
**Secondary efficacy outcomes**									
Composite events	6	11	7	1.71 (0.58–5.09)	0.334	1.16 (0.45–2.99)	0.759	1.31 (0.54–3.16)	0.549
Ischemic stroke	5	11	6	1.60 (0.49–5.23)	0.441	1.37 (0.51–3.69)	0.539	1.43 (0.56–3.66)	0.455
Hemorrhagic stroke	1	0	1	1.91 (0.12–30.63)	0.646	N/A	N/A	0.53 (0.03–8.45)	0.652
TIA	0	0	1	N/A	N/A	N/A	N/A	N/A	N/A
MI	0	0	1	N/A	N/A	N/A	N/A	N/A	N/A
Vascular death	0	2	1	N/A	N/A	1.47 (0.13–16.26)	0.751	1.06 (0.10–11.72)	0.960
Death from any cause	2	7	7	0.54 (0.11–2.61)	0.447	0.74 (0.26–2.10)	0.57	0.68 (0.25–1.84)	0.451
**Primary safety outcomes**									
Intracranial hemorrhage	1	0	1	1.91 (0.12–30.63)	0.646	N/A	N/A	0.53 (0.03–8.45)	0.652
Any bleeding	4	6	8	0.96 (0.29–3.19)	0.947	0.55 (0.19–1.58)	0.267	0.66 (0.26–1.68)	0.386

*Values are presented as number of events (percentage). HR was calculated by using Cox proportional hazards model*.

*TIA, transient ischemic attack; MI, myocardial infarction; N/A, not applicable*.

**Figure 3:**
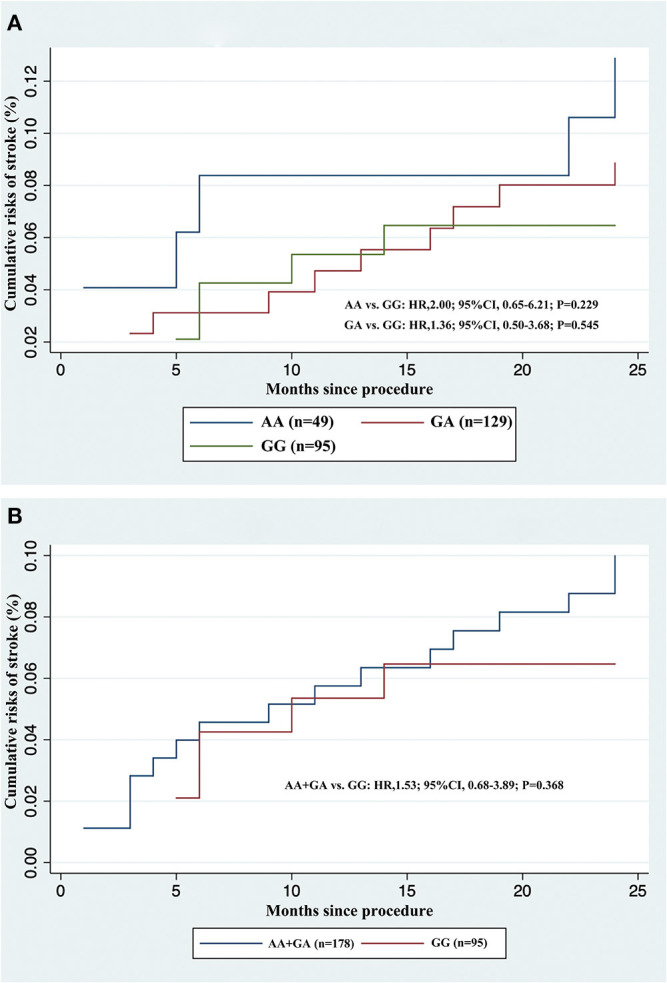
Cumulative incidences of recurrent stroke in acute minor ischemic stroke and transient ischemic attack patients treated with dual antiplatelet therapy, stratified by rs12041331 genotypes. **(A)** AA or GA carriers vs. GG wild type. **(B)** AA + GA carriers vs. GG wild type.

We did not observe any evidence of the association between PEAR1 rs12041331 genotype and cerebrovascular events in participants treated with DAPT. PEAR1 rs12041331 A allele carrier status did not result in statistically significant differences in stroke or composite clinical vascular event rates (ischemic stroke, hemorrhagic stroke, TIA, myocardial infarction, or vascular death) regardless if the individuals carried one (stroke, *P* = 0.545; composite events, *P* = 0.759; ischemic stroke, *P* = 0.539; hemorrhagic stroke, *P* = N/A; TIA, *P* = N/A; myocardial infarction, *P* = N/A; vascular death, *P* = 0.751) or two (stroke, *P* = 0.229; composite events, *P* = 0.334; ischemic stroke, *P* = 0.441; hemorrhagic stroke, *P* = 0.646; TIA, *P* = N/A; myocardial infarction, *P* = N/A; vascular death, *P* = N/A) copies of the A allele (Figure 3A and Table 2). We repeated these analyses between all AA/GA carriers and GG homozygotes and found no significant associations either (stroke, *P* = 0.368; composite events, *P* = 0.549; ischemic stroke, *P* = 0.455; hemorrhagic stroke, *P* = 0.652; TIA, *P* = N/A; myocardial infarction, *P* = N/A; vascular death, *P* = 0.960) (Figure 3B and Table 2).

### Safety Outcomes and Genotypes

The safety outcome (bleeding events) occurred in 17 patients (6.23%); among them, two were with intracranial hemorrhage. The rates of safety endpoints did not differ significantly between PEAR1 rs12041331 genotypes in participants treated with DAPT. PEAR1 rs12041331 A allele carrier status did not result in statistically significant differences in bleeding events (intracranial hemorrhage and any bleeding) regardless if individuals carried one (intracranial hemorrhage, *P* = N/A, and any bleeding, *P* = 0.267) or two (intracranial hemorrhage, *P* = 0.646, and any bleeding, *P* = 0.947) copies of the A allele (Table 2). We repeated these analyses between all AA/GA carriers and GG homozygotes and found no significant associations either (intracranial hemorrhage, *P* = 0.652, and any bleeding, *P* = 0.386; Table 2).

## Discussion

### Major Findings

In the present study, we evaluated the impact of PEAR1 rs12041331, a well-described genetic variant implicated in aspirin-related platelet function, on long-term cerebrovascular events, bleeding events, and clinical function in AMIS and TIA patients treated with DAPT. Unfortunately, it was observed in the current study that PEAR1 rs12041331 genotype was not associated with the atherothrombotic or bleeding events in minor stroke and TIA.

### Comparison With Prior Studies

PEAR1 receptor, which is highly expressed in platelets and endothelial cells, is a critical part of platelet aggregation response toward multiple agonists, and rs12041331 is a strong genetic determinant of on-treatment platelet inhibition (7, 10, 19). However, few data are reported regarding the impact of this variant on cerebrovascular event risk in AMIS and TIA patients treated with DAPT. Investigations focused on the impact of this polymorphism were mainly on cardiovascular-related diseases, but with mixed results (14). An initial study in percutaneous coronary intervention patients treated with DAPT showed that the A allele carriers of rs12041331 experienced cardiovascular events (HR = 2.62; 95% CI, 0.96–7.10; *P* = 0.059) or death (HR = 3.97; 95% CI, 1.10–14.31; *P* = 0.035) more frequently compared to GG homozygotes (20). Xu et al. assessed the AA homozygotes of PEAR1 rs12041331 and its relation to clinical outcome in over 2,400 Chinese population receiving DAPT after percutaneous coronary intervention. They found that these patients had an almost equal to three-fold increase in 30-day incidence of major adverse cardiovascular events risk compared with non-AA homozygotes (15). However, these results were not confirmed in an Egyptian acute coronary syndrome patient treated with DAPT, which reported no association with PEAR1 rs12041331 and cardiovascular risks (21). Moreover, the Aspirin in Reducing Events in the Elderly trial analyzed the relationship between PEAR1 rs12041331 and cardiovascular outcomes in a healthy elderly population with no previous atherothrombotic cardiovascular disease (14). After a median follow-up of 4.7 years, they found no significant interaction effects between the A allele carriers of rs12041331 and cardiovascular events regardless of aspirin use. This study showed that PEAR1 rs12041331 was not an important contributor to clinical events in the context of primary prevention. Consistent with its role in secondary prevention with aspirin, it does not make a contribution to clinical events in primary prevention.

At present, there are few studies on PEAR1 rs12041331 genotype in stroke. Most recently, Peng et al. explore PEAR1 rs12041331 with the platelet activity in 283 Chinese ischemic stroke patients receiving aspirin therapy, and no association was observed between platelet activity during aspirin therapy and rs12041331 (22). Zhao et al. assessed retrospectively the rs12041331 in 56 patients with recurrent ischemic stroke and 137 patients with initial stroke. They found that rs12041331 was independently associated with recurrent ischemic stroke, and the A allele showed a higher frequency than the G allele in the recurrent ischemic stroke group (17). The above-mentioned cases were the only two studies focused on the correlation between PEAR1 rs12041331 and stroke, both of which are retrospective. However, no studies have identified the impact of PEAR1 rs12041331 on the prognosis of acute stroke.

Given that aspirin and clopidogrel are the first-line treatments for the secondary prevention of atherothrombotic events in minor stroke or TIA patients and the effect of PEAR1 rs12041331 on platelet aggregation (8, 9) and in response to antiplatelet agents (10, 11), the present study has been undertaken in patients with minor stroke or TIA, which is the first research on PEAR1 rs12041331 genotype and long-term cerebrovascular outcomes conducted to date. In our investigation, we did not observe a significant association between PEAR1 rs12041331 genotype and long-term cerebrovascular events, bleeding events, and clinical function in the entire cohort. Inconsistent with those of Zhao et al. (17), our results suggested that rs12041331 genetic polymorphism is not an important contributor to clinical events in AMIS and TIA patients in the setting of secondary prevention.

### Potential Mechanism

It is necessary to clarify the potential mechanisms of clinical outcome difference response to the PEAR1 rs12041331 genotype. Individual differences in drug metabolism, response, and toxicity in humans were considered to be correlated with gene polymorphism. Besides this, ethnic differences in the PEAR1 gene polymorphism are one of the most important factors, which should be considered to explain the clinical outcome differences. Moreover, cell-specific PEAR1 methylation reveals a locus that coordinates the expressions of multiple genes (23), which may provide an explanation for the diversity of clinical events. Meanwhile, methylation is greatly influenced by environmental factors, which may constantly affect the final events. In addition, although PEAR1 rs12041331 was among the strongest determinants of platelet aggregation pre-aspirin administration, it could only account for ~15% of the total phenotypic variation in platelet function (24). In view of the fact that the occurrence of clinical events is often caused by multiple factors, exploring only one certain variable may not be enough to get a positive outcome.

### Clinical Consideration

While studies have identified some genetic determinants of inter-individual variability in on-treatment platelet inhibition (e.g., PEAR1), evidence on whether these variants have clinical value to predict vascular events remains controversial. A previous study found a dose–response relation between the expression of PEAR1 protein and the number of G alleles at rs12041331 in response to several agonists in human platelets (19). However, most cardiovascular studies on PEAR1 rs12041331 genotype have found that rs12041331 A allele is more prone to cardiovascular events. As a preliminary observational study, we found no impact of PEAR1 rs12041331 on the prognosis of minor stroke and TIA. Due to the low number of patients and events, these results should also be interpreted with caution, and further analysis of other large research is therefore warranted.

### Strengths and Limitations

This is a study that assessed the association of PEAR1 rs12041331 genetic polymorphism and the long-term cerebrovascular events, bleeding events, and clinical functions in patients with minor stroke or TIA. The results from this study will provide a reference for the relationship between genetic susceptibility of anti-platelet aggregation therapy and cerebrovascular risks in clinical practice. This study has some limitations that should be highlighted. First, the data that we collected were from a single center, so the sample size was not large enough, which might limit the generalizability of our findings. Second, our study is limited to the reporting of long-term clinical outcomes, lacking on-treatment platelet reactivity, which can more intuitively reflect the risks of thrombosis. Third, PEAR1 rs12041331 was reported to be more associated with platelet aggregation of aspirin, but the subjects of our study were treated with DAPT, not aspirin alone, so we cannot exclude the effects of other pivotal genes related to clopidogrel on outcomes, such as CYP2C19 polymorphisms, which are fully known to affect the platelet reactivity of clopidogrel. Future studies, after adjusting for other gene polymorphisms like CYP2C19, are needed to explore the role of PEAR1 rs12041331.

## Conclusion

We could not replicate the previous findings suggesting that A allele carriers of PEAR1 rs12041331 were an important genetic determinant of clinical atherothrombotic or bleeding events. Our data do provide robust evidence that genetic variation in PEAR1 rs12041331 does not contribute to atherothrombotic or bleeding risk in minor stroke and TIA patients treated with DAPT.

## Data Availability Statement

The datasets presented in this study can be found in online repositories. The names of the repository/repositories and accession number(s) can be found in the article/Supplementary Material.

## Ethics Statement

The studies involving human participants were reviewed and approved by Ethics Committee of Yangpu Hospital, Tongji University School of Medicine. The patients/participants provided their written informed consent to participate in this study.

## Author Contributions

Y-ML and Y-HY conceived and designed this study. X-GZ, Q-QF, S-WC, S-SJ, and JX were involved in the acquisition and interpretation of the data. X-GZ, J-YG, and Q-QF wrote the manuscript with contributions from all the authors. Y-MK and Y-HY refined the manuscript. All the authors read and approved the final manuscript.

## Conflict of Interest

The authors declare that the research was conducted in the absence of any commercial or financial relationships that could be construed as a potential conflict of interest.
